# Determinants of Thermostability in Serine Hydroxymethyltransferase Identified by Principal Component Analysis

**DOI:** 10.1038/srep46463

**Published:** 2017-04-19

**Authors:** Fei Leng, Lu-Yun Wu, Chang Lu, Xian-Ming Pan

**Affiliations:** 1 Key Laboratory of Bioinformatics, Ministry of Education, School of Life Sciences, Tsinghua University, Beijing, 100084, China

## Abstract

Protein thermostability has received growing attention in recent years. Little is known about the determinants of thermal resistance in individual protein families. However, it is known that the mechanism is family-dependent and not identical for all proteins. We present a multivariate statistical analysis to find the determinants of thermostability in one protein family, the serine hydroxymethyltransferase family. Based on principal component analysis, we identified three amino acid fragments as the potential determinants of thermostability. The correlation coefficients between all the putative fragments and the protein thermostability were significant according to multivariable linear regression. Within the fragments, four critical amino acid positions were identified, and they indicated the contributions of Leu, Val, Lys, Asp, Glu, and Phe to thermostability. Moreover, we analyzed the insertions/deletions of amino acids in the sequence, which showed that thermophilic SHMTs tend to insert or delete residues in the C-terminal domain rather than the N-terminal domain. Our study provided a promising approach to perform a preliminary search for the determinants of thermophilic proteins. It could be extended to other protein families to explore their own strategies for adapting to high temperature.

Serine hydroxymethyltransferase (SHMT) is a pyridoxal 5′-phosphate (PLP)-dependent enzyme that catalyzes the reversible transfer of serine to glycine, transferring Cβ to tetrahydropteroylglutamate (H_4_PteGlu) and forming 5,10-methylene-H_4_PteGlu^1^. SHMT is one of very few enzymes to contain tightly-bound PLP as a cofactor^2^, and because it plays a key role in amino acid metabolism and the biosynthesis of most neurotransmitters, it has been considered a potential target for the development of anticancer and antimicrobial agents^3^,^4^,^5^.

SHMT is found in all living organisms, leading to its broad adaptation in various environments, including extremely high temperatures above 75 °C^6^. Compared with mesophilic SHMTs, thermophilic SHMTs share a nearly 80% amino acid sequence identity. Their 3D structures are highly conserved, and very few structural changes have occurred during evolution^1^. Although they have adapted to different temperatures, the enzymatic activities of mesophilic and thermophilic SHMTs are not significantly different. Because of this, we can find and create enzymes that can withstand harsh conditions for industrial applications^7^,^8^ and make a greater impact on the field of biocatalysis and cellular metabolism.

In the past few decades, considerable attention has been paid to thermophilic proteins, and many sequence and structural factors have been correlated with protein thermostability. One of the most noteworthy features is the amino acid composition of the sequence, especially the charged and hydrophobic residues^9^. Likewise, the substitution of certain amino acids may enhance or decrease the thermostability of a protein^10^,^11^. Increasing the number of salt bridges and hydrogen bonds, applying tighter hydrophobic packing, and decreasing the unfolding entropy also contribute to protein thermostability^12^,^13^,^14^,^15^. However, many features show a strong protein family dependence and are not useful for all proteins^16^,^17^,^18^,^19^. The contribution of these factors to protein thermostability is still debatable, and some features even make negative contributions. Additionally, amino acids can be affected by neighboring sites within their local environment.

A better understanding of protein thermostability can be achieved by discriminating between the mesophilic and thermophilic proteins within a family and predicting the determinants of the protein thermostability of the family. Accordingly, 378 SHMT sequences were included in the dataset, including 305 mesophilic SHMTs, whose OGTs are higher than 20 °C and lower than 40 °C, and 73 thermophilic SHMTs, whose OGTs are higher than 50 °C. Then, we selected several amino acid features that may be related to protein thermostability according to previous studies. In this study, principal component analysis (PCA) was introduced to predict the determinants of protein thermostability, and the accuracy of the prediction was evaluated by a multivariable linear regression (MLR) test. We found three amino acid fragments to be the determinants of thermostability in SHMT. Moreover, we analyzed the insertion and deletion of amino acids and found that they rarely occurred in the PLP-binding domain of the chain. Thermophilic SHMTs tend to insert or delete residues in the C-terminal sub-domain and insert residues at the N-terminal domain, which indicates the importance of the C-terminal domain to the protein thermostability.

## Result

### SHMT thermostability source identification

**Figure 1:**
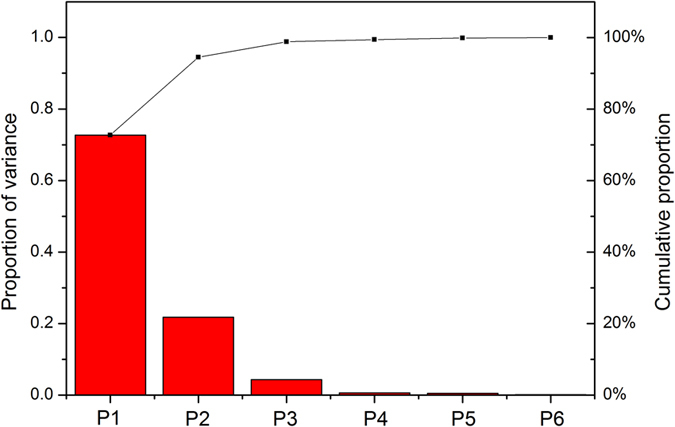
Variance of principal components. The histogram indicates the variance proportion of each principal component from P1 to P6. The line chart indicates the cumulative proportions of the six principal components.

Previous studies have revealed that a variety of factors contribute to the protein thermostability, and most of these factors are correlated. In this work, we used PCA to reduce the original number of factors that were reported to be related to protein thermostability. According to previous research^20^,^21^,^22^, six physico-chemical properties of amino acids are considered the original factors. We extracted indexes from the original factors and estimated the correlations between thermostability and the extracted indexes. As shown in Fig. 1, two principal components (PCs), P1 and P2, are shown to represent 73% and 22% of the variance of the original factors, and they can explain more than 90% of the variance together. From the variance interpreted by the principal components, the proportion of the variance interpreted by the first PC is 73%, and according to the loadings of the first PC, the weighting coefficient of the variable side chain is the largest. In the second PC, the proportion of variance is 22%, and the weighting coefficient of the variable hydrophobicity is the largest. By these, we can speculate that the side chain and hydrophobicity are the most important factors and could explain most of the variance. We used these two PCs as SHMT thermostability sources, termed factor loadings (FLs). PCs that had a standard deviation less than 1 (from P2 to P6) were neglected as they were less important.

### Detection of crucial amino acid positions

**Figure 2:**
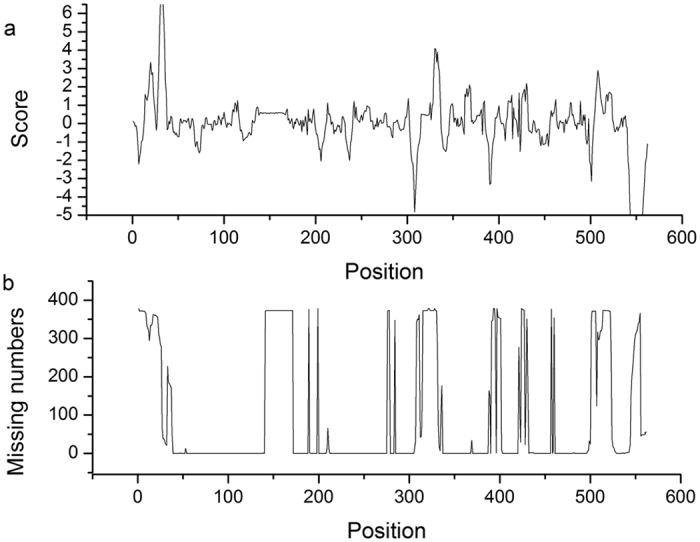
(**a**) Scoring trend of the amino acid fragments. The X axis indicates the positions of the fragments along the multiple alignment of the SHMTs sequence, and the Y axis indicates the score of the fragment at each position. (**b**) Trend of the missing numbers at all positions. The X axis indicates the position along the multiple alignment, and the Y axis indicates the numbers of sequences with missing residues at each position.

Our method used PCA to determine the variation between meso- and thermophilic SHMTs for different amino acid fragments, and each fragment received a score indicating the extent of variation. The trend of the scores of all amino acid fragments is shown in Fig. 2a. It is obvious that fragments 30–35 and 330–333 are outstanding compared to the rest of the fragments. However, we note that positions in these sections are deleted in more than two hundred SHMTs, and this may affect the scores of the fragments. As a result, we calculated the missing numbers at each site in the multiple alignment. Figure 2b shows the trend of the missing statistics, which represents insertions/deletions at each site. When the missing number equals zero, the insertion or deletion of an amino acid is unlikely at that site. In this situation, a point mutation of an amino acid is the only way to alter the properties of that site.

**Figure 3:**
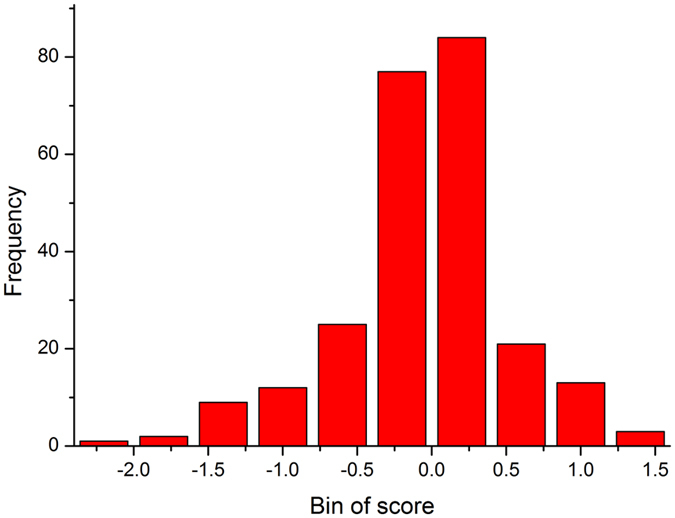
Frequency distribution of scores. The X axis is the score with a bin of 0.5, and the Y axis is the frequency of each bin score.

To rule out underlying factors that are introduced by the insertion or deletion of amino acids, we identified and removed fragments that contained positions with missing numbers. The scores of the remaining amino acid fragments were calculated, and Fig. 3 shows the frequency distribution of the scores. Fragments with a score >1.2 can be easily discriminated with a p < 0.01. On this basis, three amino acid fragments were found to be potential determinants. To validate the thermostability contribution of the three candidate fragments, multivariable linear regression (MLR) was used to analyze the relationship between the physico-chemical features of the fragments and the thermostability of the SHMTs.

As shown in Table 1, the first fragment is from residues 115 to 121 in the multiple alignments. This fragment was localized in the PLP-binding region of the chain. The score of this fragment was 1.26, and the p-value was 0.009. According to MLR, the coefficient of multiple correlation was 0.59 (p < 0.001). Of the six physico-chemical features probed, the hydrophobicity, polarity and side chain were significant impact factors. The second fragment was from residues 242 to 248 in the multiple alignments and was localized in the PLP-binding region of the chain. The score of this fragment was 1.22, and the p-value was 0.01. According to MLR, the coefficient of multiple correlation was 0.57 (p < 0.001). Of the six physico-chemical features tested, the molecular weight, side chain and buriability were significant impact factors. The third fragment was from residues 462 to 468 in the multiple alignment and was localized in the C-terminal region of the chain. The score of this fragment was 1.31, and the p-value was 0.007. According to MLR, the coefficient of multiple correlation was 0.58 (p < 0.001). Of the six physico-chemical features, the hydrophobicity, side chain and buriability were significant impact factors.

**Figure 4:**
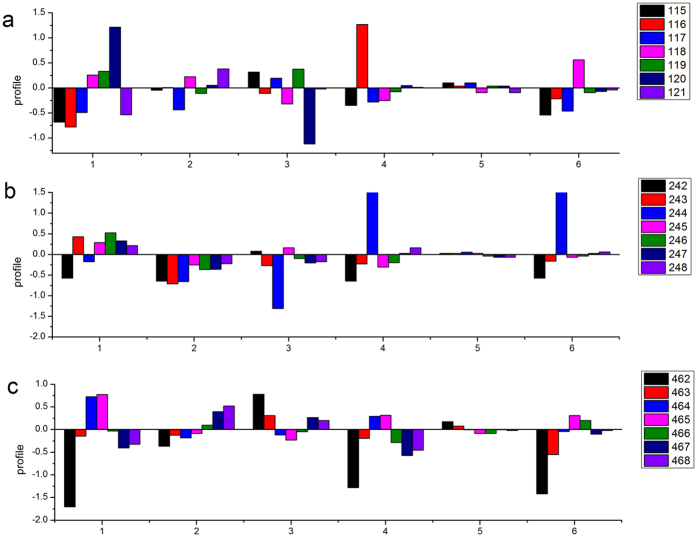
Profile of the differences in the physico-chemical properties of thermophilic SHMTs and mesophilic SHMTs. (**a)** Fragment of residues 115–121; (**b)** fragment of residues 242–248; and (**c)** fragment of residues 462–468. The X axis indicates the six physico-chemical properties: 1, hydrophobicity; 2, molecular weight; 3, polarity; 4, side chain; 5, flexibility; and 6, buriability. The Y axis indicates the profile of the differences between the thermophilic SHMTs and mesophilic SHMTs.

As candidate fragments for thermostability in SHMTs, single amino acid positions within the three fragments were studied to determine the ones that were important. By analyzing the fragment from residues 115 to 121, two critical positions, 116 and 120, were identified (Fig. 4a). The first position was position 116, and thermophilic SHMTs showed a significant increase in the side-chain contributions to the stability. The amino acid position 120 of the thermophilic SHMTs similarly showed a strong increase in hydrophobicity and a strong decrease in the polarity contribution to the stability. At position 116, the content of Lys was 29% in the thermophilic SHMTs and 7% in the mesophilic SHMTs. At position 120, the content of Leu was 25% in the thermophilic SHMTs and 0.3% in the mesophilic SHMTs.

For the 242 to 248 fragment, position 244 was the most crucial. At this position in the thermophilic SHMTs, there was a significant increase in side chains and buriability and a strong decrease in polarity with respect to the mesophilic SHMTs (Fig. 4b). The thermophilic SHMTs showed a higher content of the hydrophobic residue Val (32%) and aromatic residue Phe (52%) compared to the mesophilic SHMTs (0.7% and 45%, respectively).

An analysis of the fragment of 462 to 468 showed that position 462 was very important (Fig. 4c). This site showed a significant decrease in hydrophobicity, side chains and buriability in the thermophilic SHMTs. Amino acid composition analysis revealed a remarkably higher content of the charged residues Asp and Glu (29%) in thermophilic SHMTs with respect to mesophilic SHMTs (1%).

**Figure 5:**
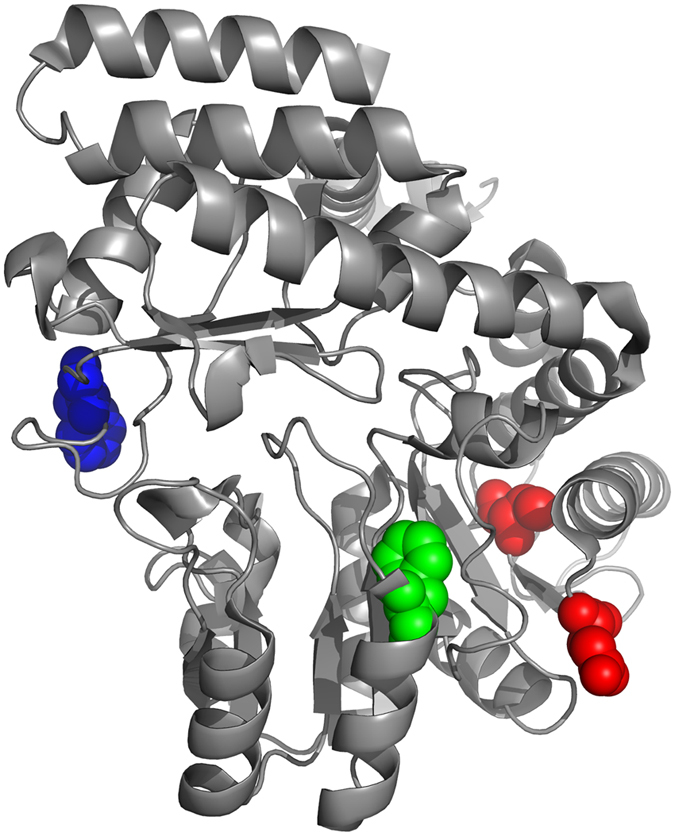
3D structure of the thermophilic Bacillus stearothermophilus. (Protein Data Bank accession number 1KKJ.) The regions in color indicate the 4 candidate amino acid positions for thermal stability (red for positions 116 and 120, green for position 244, and blue for position 462; the positions are converted according to the multiple alignment).

The structure of SHMT is presented in Fig. 5. As we can see, positions 116 and 120 are in the loop region and at both ends of a β-sheet. They are located in a cluster of hydrophobic residues, which is essential for protein folding. Position 244 is located in a β-sheet and is buried in a hydrophobic core, which was demonstrated to be important for the SHMT stability. Position 462 is in the loop region and appeared to be important for the FTHF binding, which could change the protein conformation^23^.

### Result of molecular simulation

**Figure 6:**
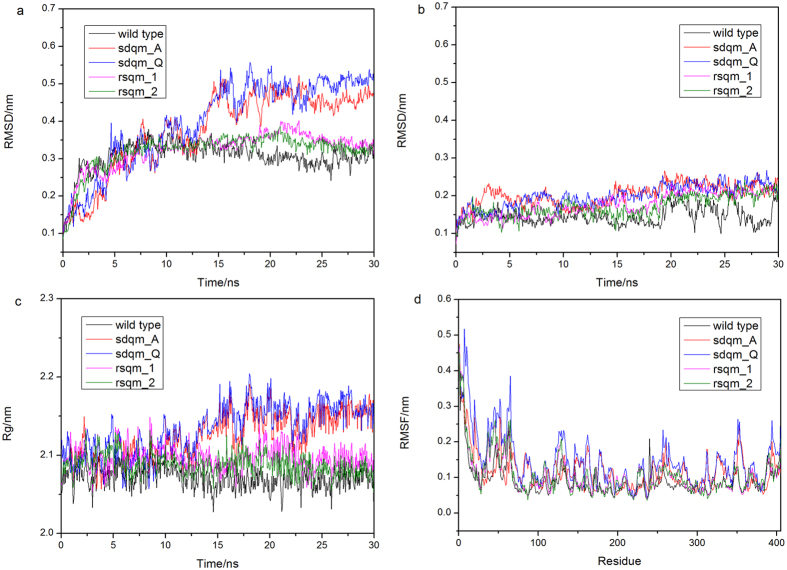
RMSD as a function of the simulation time averaged over 30 ns (**a**) at 350 K and (**b**) at 300 K. (**c**) Rg as a function of the simulation time averaged over 30 ns at 350 K. (**d**) RMSF as a function of the simulation time averaged over 30 ns at 350 K. The black line indicates the wild type, the red line and blue line indicate the site-directed-quadruple-mutants (sdqm_A and sdqm_Q), the magenta line and the green line indicate the randomly-selected-quadruple-mutants (rsqm_1 and rsqm_2).

Molecular simulation has been used in previous studies to analyze the protein thermostability and unfolding behaviors, where the dynamic behavior of the proteins under various simulation temperatures have been utilized as a criterion for the protein thermostability^24^,^25^. In our research, to validate our method, we performed molecular simulations to explore the differences in the thermal stability between the wild type of thermophilic SHMT and those with mutations in the key positions or in the randomly selected positions. Four mutants were designed for comparison, including two site-directed-quadruple-mutants (sdqm_A and sdqm_Q) and two randomly-selected-quadruple-mutants (rsqm_1 and rsqm_2). Simulations of wild type SHMT and four mutants were carried out for 30 ns at 350 K. We analyzed the root mean square deviation (RMSD), root mean square fluctuation (RMSF) and radius of gyration (Rg) for these proteins. The RMSD and Rg as a function of the simulation time for those five proteins are plotted in Fig. 6a and c. The wild type and randomly-selected-quadruple-mutants remained relatively stable, although the randomly-selected-quadruple-mutants showed a slightly greater fluctuation than the wild type. For both of the two site-directed-quadruple-mutants, Rg starts to increase after 12 ns and RMSD then increases immediately. As well as change in conformation, flexibility of the structure is also altered by site-directed mutations. We calculated the RMSF to measure overall flexibility and observed a significant increase RMSF in both of the two site-directed-quadruple-mutants (Fig. 6d). It can be noticed that the site-directed-quadruple-mutants affect the neighboring residues, indicating a gain of flexibility due to mutations. As a control, the five proteins were simulated for 30 ns at 300 K. As shown in Fig. 6b, the structures of the wild type and mutants are stable and the RMSDs of the five proteins remain unchanged throughout the 30-ns simulation. These are consistent with our prediction that mutations in the determinant amino acids would decrease the thermostability of the protein.

### Detection of amino acid insertions and deletions

Positions with non-zero missing numbers may lead to an incorrect prediction of insertions or deletions using the above method. As a result, we performed a separate statistical analysis for amino acid insertions and deletions.

In multiple alignments, there were 206 positions with missing numbers not equal to zero, and an amino acid could be inserted or deleted at these positions. To probe the effect of an insertion or deletion on the SHMT thermostability, we calculated the missing rate of each position in the mesophilic and thermophilic SHMTs, and then we obtained the MRT value (missing rate of thermostability) to evaluate the thermal contribution of the insertion or deletion at each position.

A statistical analysis showed that more than 60% of MRTs are located near a positive with a zero value. The Supplementary information shows the detailed data (see Supplementary Table S1). Here, we focus on positions with a high absolute value of the MRT: more than 0.5 or less than −0.5. An insertion or deletion at these positions would make sense for the SHMT thermostability.

**Figure 7:**
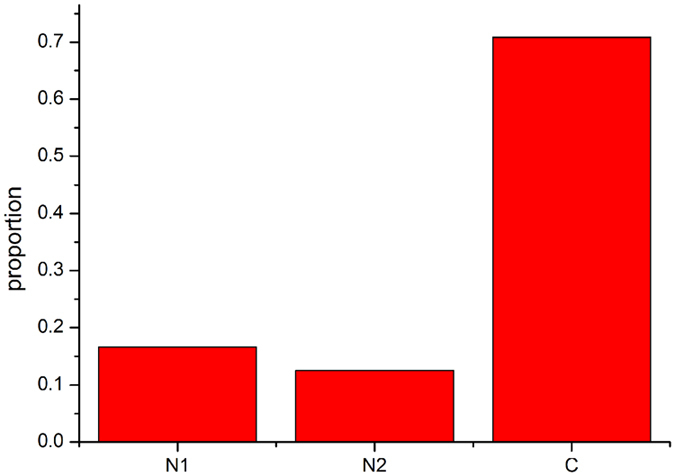
Deletion status of the three domains. N1 indicates the N-terminal domain. N2 indicates the PLP-binding domain, and C indicates the C-terminal domain.

According to the region of the chain, the positions were divided into three groups: N1MRT, located in the N-terminal sub-domain; N2MRT, located in the PLP-binding domain; and CMRT, located in the C-terminal domain. Figure 7 shows that N2MRT (insertion and deletion in the PLP-binding domain) contributes minimally to thermostability, and almost 90% of the high absolute values of the MRTs were located in the two ends of the sequence, the N-terminal sub-domain and the C-terminal domain, with more than 70% in the C-terminal sub-domain. This indicates that for thermophilic SHMTs, most insertions or deletions happen in the C-terminal domain.

## Discussion

The mechanism by which proteins adapt to extreme temperature has been studied for several decades. By comparing the sequences and structural characteristics of thermophilic proteins with those of mesophilic proteins, some features were determined to be related to the protein thermostability. Of these, the composition and substitution of certain amino acids were most noticeable, such as charged and hydrophobic residues. However, it is still unclear how to identify specific positions responsible for protein thermostability, which would be very useful for the industrial application of thermophilic enzymes. Additionally, the contribution of some factors to protein thermostability may vary, and they can even play opposing roles when their positions change. Thus, it is necessary to find specific positions that are responsible for protein thermostability.

According to previous research, each protein family seems to have its own strategy to adapt to high or low temperatures, and the factors to which thermostability may be attributed may not be appropriate to all proteins. This indicates that thermostability depends not only on specific factors but also the local environment. In this study, we analyzed one typical family, SHMTs. Because of their ubiquitous nature and critical metabolic role, SHMTs represent an ideal paradigm of enzymes to study protein adaptations for extreme environments. Based on the sequences of SHMTs from organisms with known optimal growth temperatures (OGTs), we proposed a statistical approach for predicting the determinant of thermostability in SHMTs.

PCA detected three amino acid fragments as putative substitution positions affecting protein stability. Two of the fragments were located in the PLP-binding domain. As PLP has been shown to play a significant role in the functional activity and stability of SHMTs, substitutions in the region would have significant consequences in the structure and stability of SHMTs^26^. All of the putative positions are in the middle of a loop and point to the interior of the protein and are a part of a hydrophobic fragment^27^. Mutations within these positions would alter the structural integrity and result in a change in folding. For example, changes in the conserved sequence may be exploited for designing SHMT drugs^28^. When examining the determinate fragments, we detected four amino acid positions critical for thermostability. The identification of the residue that most frequently occurs in each of the four critical positions yielded three types of residues as the consensus sequence of thermophilic SHMTs: the hydrophilic residues Leu and Val; the charged residues Lys, Asp and Glu; and the aromatic residue Phe. The contents of these residues in thermophilic SHMTs are significantly higher than those in mesophilic SHMTs. In general, they would lead to increasing hydrophobicity, electrostatic charge, cation-π and packing while decreasing the flexibility in thermophilic SHMTs, which is consistent with previous studies^29^,^30^,^31^.

By examining the missing numbers of amino acids, we found that thermophilic proteins tend to insert or delete residues in the C-terminal domain. In a previous experiment study, it has been demonstrated that the C-terminal domain plays a vital role in the stabilization of the quaternary structure and is associated with unfolding processes^32^. Additionally, hydrophobic interactions at intersubunit interfaces have been proposed to contribute to the thermostability of thermophilic proteins^33^,^34^. In this light, the C-terminal domain may be exploited to improve the chemical-physical stability of SHMTs.

Based on the study of SHMTs, this paper improves the understanding of the protein thermostability in an individual family. Hydrophobic residues, charged residues and aromatic residues are shown to have significant effects on the protein thermostability. On the other hand, our study indicates the importance of the C-terminal of SHMT to the protein stability. These findings in SHMTs are expected to lead to improved understanding of the biochemical and structural properties of thermophilic proteins and the development of novel catalytic function and enzyme production.

## Methods

### Dataset construction

SHMT sequences were collected from Swiss-Prot (http://www.ebi.ac.uk/uniprot/). Information about the organisms was obtained from NCBI, and those with known optimal growth temperatures (OGTs) were chosen for the dataset. 378 SHMT sequences were included in the dataset, including 305 mesophilic SHMTs, whose OGTs are higher than 20 °C and lower than 40 °C and 73 thermophilic SHMTs, whose OGTs are higher than 50 °C. The length of the sequence ranged from 406 to 497.

### Data pretreatment

To obtain the standardized position, Bacillus stearothermophilus SHMT (bsSHMT) was joined for an alignment. The sequences of the SHMTs in the dataset and bsSHMT were aligned using ClustalX^35^, and a multiple alignment of mesophilic SHMTs and thermophilic SHMTs was produced respectively. As a template, bsSHMT was concluded in both of the groups. To assess the alignment result, we calculated the confidence scores using GUIDANCE^36^. The scores of the mesophilic SHMTs and thermophilic SHMTs are 0.96 and 0.93, which proved a good alignment. Then, based on the position of the bsSHMT, all the multiple alignments were merged. Finally, according to the model of bsSHMT, the positions of the SHMTs in the dataset were divided into two domains^37^, the N-terminal domain (residues 1–370) and the C-terminal domain (residues 371–562). In the subsequent steps, the N-terminal domain was divided into two sub-domains, a small N-terminal sub-domain (residues 1–111) and a PLP-binding domain (residues 112–370). Briefly, three domains were generated.

### Physico-chemical properties of amino acids

Six physico-chemical properties of amino acids were selected as factors for linear discriminant analysis, including three general properties (hydrophobicity, molecular weight and polarity) and three specific properties (side chain, flexibility and buriability) based on previous studies^20^,^21^,^22^. According to the AAindex database^38^, the AAindex codes of the 6 properties were set as COWR900101, FASG760101, GRAR740102, TAKK010101, VINM940101 and ZHOH040103.

For each sequence, an amino acid was moved from the N-terminal region to the C-terminal region with a sliding window of 7 residues and a step of one residue^39^. The average of the six properties over the seven residues in a fragment was calculated.

If we define s as the length of the multiple alignment sequence, there will be s-6 fragments. If i was the position in the sequence, p(i) is the property index of position i. For a given sequence of a thermophilic SHMT, the property index of each fragment was defined as Pt(i), which was calculated as follows:


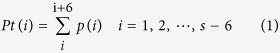


Thus, we can obtain the average of the Pt(i) of all thermophilic SHMTs, 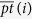.

For a given sequence of a mesophilic SHMT, the property index of each fragment was defined as Pm(i), which was calculated as follows:


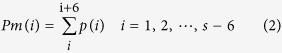


Thus, we can obtain the average of Pm(i) of all mesophilic SHMTs, 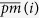.

The difference in the property index between thermophilic SHMTs and mesophilic SHMTs was regarded as the thermostability contribution of the property from the fragment. For a given fragment, it was calculated as follows:


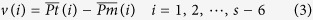


### Establishment of a mathematical model

According to the previous equation, there were 383 amino acid fragments. We considered all of the 383 fragments as samples, and each sample has six factors, *V*_1_, *V*_2_, …, *V*_6_. Thus, the original database can be defined as


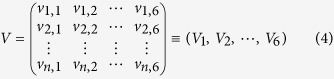


where 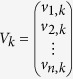 *k* = 1, 2, …, 6

V_i_ is an n-dimensional vector, n is the number of fragments, and k is the index of the factor.

According to matrix V, a linear combination can be made as


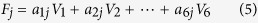


where F_j_ is eigenvector j. a_1j_ to a_6j_ are the coefficients of their respective variables, and the values of a_1j_^2^ + a_2j_^2^ + … + a_6j_^2^ are set as 1. F_j_ is decreasing as j increases.

### PCA analysis

All values in the matrix were standardized using R software. A smaller set of independent variables, principal components (PCs), were generated by PCA, explaining the variance of the whole set. To increase the reliability of our interpretation, the PCs were rotated using a varimax rotation to minimize the complexity of the components. Using PCs with a standard deviation greater than 1, new variables, known as factor loadings (FLs), were obtained. We chose FLs with a cumulative variance contribution rate larger than 80% for the score calculation, and the score of the fragment in position i was


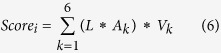


where


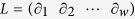



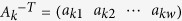


where L is a one-dimensional vector, A_k_ is w-dimensional vectors, and w is the number of FLs that we chose.

### Simulation procedures

The wild-type structure was obtained from the crystal structure of the thermophilic SHMT (PDB code: 1KKJ). The positions of original sequence were changed after the alignment, and residues 85, 89, 180 and 351 were in positions 116, 120, 244 and 462, respectively, in the multiple alignment. Accordingly, for the original sequence of the wild type, 85, 89, 180 and 351 were the four crucial residues we determined. Using PYMOL (http://www.pymol.org), two site-directed-quadruple-mutants were constructed, including E85A, V89A, F180A and F351A (sdqm_A) and E85Q, V89Q, F180Q, F351Q (sdqm_Q). For comparison, two randomly-selected-quadruple-mutants were constructed, including Y61A, V139A, P258A, N310A (rsqm_1) and G83A, T111A, D153A, F270A (rsqm_2). All the generated structures were solvated and neutralized in TIP3P water with a minimum of 10 Å between the model and the wall of the box. Simulations were performed using NAMD 2.9 with periodic boundary conditions (PBC) applied^40^ and a CHARMM36 force field^41^. The particle mesh Ewald method was applied, and the van der Waals interactions cutoff was set at 12 Å. The pressure was held at 1 atm. Four MD simulations were carried out at 300 K or 350 K for both wild-type and mutated models. All systems followed a 3-step pre-equilibration totaling 1.4 ns, and then the MD simulations were carried out for the total times listed in Table 2. The simulations were analyzed with GROMACS 5.2^42^ and viewed using VMD^43^.

## Additional Information

**How to cite this article:** Leng, F. *et al*. Determinants of Thermostability in Serine Hydroxymethyltransferase Identified by Principal Component Analysis. *Sci. Rep.* **7**, 46463; doi: 10.1038/srep46463 (2017).

**Publisher's note:** Springer Nature remains neutral with regard to jurisdictional claims in published maps and institutional affiliations.

## Supplementary Material

Supplementary Information

## Figures and Tables

**Table 1:** Potential determinant fragments.

Position	region	Score	R	factors
115–121	PLP-binding	1.26	0.59	hydrophobicity, polarity and side chain
242–248	PLP-binding	1.22	0.57	molecular weight, side chain and buriability
462–468	C-terminal	1.31	0.58	hydrophobicity, side chain and buriability

R: coefficient of multiple correlation.

**Table 2:** Setup of the simulations.

Protein	Wild-type		Mutant	
Temperature (K)	300	350	300	350
Counterions (Na^+^)	8	8	7	7
Box size (Å^3^)	80 × 100 × 90	80 × 100 × 90	80 × 100 × 90	80 × 100 × 90
Ensemble	NTP	NTP	NTP	NTP
Total time (ns)	30	30	30	30

## References

[b1] Angelaccio S. Extremophilic SHMTs: From Structure to Biotechnology. Biomed Res. Int. 2013, 851428 (2013). 23841096 10.1155/2013/851428 PMC3697235

[b2] Singh R. . Chemogenomics of Pyridoxal 5′-Phosphate Dependent Enzymes. J Enzyme Inhib Med Chem. 28, 183–194 (2013). 22181815 10.3109/14756366.2011.643305

[b3] Amadasi A. . Pyridoxal 5′-Phosphate Enzymes as Targets for Therapeutic Agents. Curr. Med. Chem. 14, 1291–1324 (2007). 17504214 10.2174/092986707780597899

[b4] Daidone F. . In Silico and in Vitro Validation of Serine Hydroxymethyltransferase as a Chemotherapeutic Target of the Antifolate Drug Pemetrexed. Eur. J. Med. Chem. 46, 1616–1621 (2011). 21371789 10.1016/j.ejmech.2011.02.009

[b5] di Salvo M. L., Contestabile R., Paiardini A. & Maras B. Glycine Consumption and Mitochondrial Serine Hydroxymethyltransferase in Cancer Cells: The Heme Connection. Med. Hypotheses. 80, 633–636 (2013). 23474074 10.1016/j.mehy.2013.02.008

[b6] Ide H. . Purification of Serine Hydroxymethyltransferase From Bacillus Stearothermophilus with Ion-Exchange High-Performance Liquid Chromatography. J Chromatogr. 596, 203–209 (1992). 1400837 10.1016/0021-9673(92)85008-h

[b7] Yeoman C. J. . Thermostable Enzymes as Biocatalysts in the Biofuel Industry. Adv. Appl. Microbiol. 70, 1–55 (2010). 20359453 10.1016/S0065-2164(10)70001-0 PMC4561533

[b8] Chantasingh D., Pootanakit K., Champreda V., Kanokratana P. & Eurwilaichitr L. Cloning, Expression, and Characterization of a Xylanase 10 From Aspergillus Terreus (BCC129) in Pichia Pastoris. Protein Expr Purif. 46, 143–149 (2006). 16275128 10.1016/j.pep.2005.09.013

[b9] Yang H. M., Yao B. & Fan Y. L. Recent Advances in Structures and Relative Enzyme Properties of Xylanase. Sheng Wu Gong Cheng Xue Bao. 21, 6–11 (2005). 15859321

[b10] McDonald J. H., Grasso A. M. & Rejto L. K. Patterns of Temperature Adaptation in Proteins From Methanococcus and Bacillus. Mol. Biol. Evol. 16, 1785–1790 (1999). 10605119 10.1093/oxfordjournals.molbev.a026090

[b11] Lin Y. S. Using a Strategy Based On the Concept of Convergent Evolution to Identify Residue Substitutions Responsible for Thermal Adaptation. Proteins. 73, 53–62 (2008). 18384082 10.1002/prot.22049

[b12] Delboni L. F. . Crystal Structure of Recombinant Triosephosphate Isomerase From Bacillus Stearothermophilus. An Analysis of Potential Thermostability Factors in Six Isomerases with Known Three-Dimensional Structures Points to the Importance of Hydrophobic Interactions. Protein Sci. 4, 2594–2604 (1995). 8580851 10.1002/pro.5560041217 PMC2143043

[b13] Honig B., Ray A. & Levinthal C. Conformational Flexibility and Protein Folding: Rigid Structural Fragments Connected by Flexible Joints in Subtilisin BPN. Proc Natl Acad Sci USA 73, 1974–1978 (1976). 1064867 10.1073/pnas.73.6.1974 PMC430430

[b14] Chan C. H. . Relationship Between Local Structural Entropy and Protein Thermostability. Proteins. 57, 684–691 (2004). 15532068 10.1002/prot.20263

[b15] Lee C. W., Wang H. J., Hwang J. K. & Tseng C. P. Protein Thermal Stability Enhancement by Designing Salt Bridges: A Combined Computational and Experimental Study. PLoS One 9, e112751 (2014). 25393107 10.1371/journal.pone.0112751 PMC4231051

[b16] Szilagyi A. & Zavodszky P. Structural Differences Between Mesophilic, Moderately Thermophilic and Extremely Thermophilic Protein Subunits: Results of a Comprehensive Survey. Structure. 8, 493–504 (2000). 10801491 10.1016/s0969-2126(00)00133-7

[b17] Vieille C. & Zeikus G. J. Hyperthermophilic Enzymes: Sources, Uses, and Molecular Mechanisms for Thermostability. Microbiol Mol Biol Rev. 65, 1–43 (2001). 11238984 10.1128/MMBR.65.1.1-43.2001 PMC99017

[b18] Gianese G., Bossa F. & Pascarella S. Comparative Structural Analysis of Psychrophilic and Meso- and Thermophilic Enzymes. Proteins. 47, 236–249 (2002). 11933070 10.1002/prot.10084

[b19] Beeby M. . The Genomics of Disulfide Bonding and Protein Stabilization in Thermophiles. PLoS Biol. 3, e309 (2005). 16111437 10.1371/journal.pbio.0030309 PMC1188242

[b20] Zhou H. Y. & Zhou Y. Q. Quantifying the Effect of Burial of Amino Acid Residues On Protein Stability. Proteins-Structure Function and Genetics 54, 315–322 (2004). 10.1002/prot.10584 14696193

[b21] Takano K. & Yutani K. A New Scale for Side-Chain Contribution to Protein Stability Based On the Empirical Stability Analysis of Mutant Proteins. Protein Engineering 14, 525–528 (2001). 11579219 10.1093/protein/14.8.525

[b22] Menendezarias L. & Argos P. Engineering Protein Thermal-Stability - Sequence Statistics Point to Residue Substitutions in Alpha-Helices. J. Mol. Biol. 206, 397–405 (1989). 2716053 10.1016/0022-2836(89)90488-9

[b23] Trivedi V. . Crystal Structure of Binary and Ternary Complexes of Serine Hydroxymethyltransferase From Bacillus Stearothermophilus - Insights Into the Catalytic Mechanism. J. Biol. Chem. 277, 17161–17169 (2002). 11877399 10.1074/jbc.M111976200

[b24] Caflisch A. & Karplus M. Acid and Thermal-Denaturation of Barnase Investigated by Molecular-Dynamics Simulations. J. Mol. Biol. 252, 672–708 (1995). 7563082 10.1006/jmbi.1995.0528

[b25] Xu X., Su J., Chen W. & Wang C. Thermal Stability and Unfolding Pathways of Sso7d and its Mutant F31A: Insight from Molecular Dynamics Simulation. J. Biomol. Struct. Dyn. 28, 717–727 (2011). 21294584 10.1080/07391102.2011.10508601

[b26] Cai K., Schirch D. & Schirch V. The Affinity of Pyridoxal 5′-Phosphate for Folding Intermediates of Escherichia Coli Serine Hydroxymethyltransferase. J. Biol. Chem. 270, 19294–19299 (1995). 7642604 10.1074/jbc.270.33.19294

[b27] Fu T. F., Boja E. S., Safo M. K. & Schirch V. Role of Proline Residues in the Folding of Serine Hydroxymethyltransferase. J. Biol. Chem. 278, 31088–31094 (2003). 12773539 10.1074/jbc.M303779200

[b28] Chaturvedi S. & Bhakuni V. Unusual Structural, Functional, and Stability Properties of Serine Hydroxymethyltransferase From Mycobacterium Tuberculosis. J. Biol. Chem. 278, 40793–40805 (2003). 12913008 10.1074/jbc.M306192200

[b29] Paiardini A., Gianese G., Bossa F. & Pascarella S. Structural Plasticity of Thermophilic Serine Hydroxymethyltransferases. Proteins. 50, 122–134 (2003). 12471605 10.1002/prot.10268

[b30] Siglioccolo A., Bossa F. & Pascarella S. Structural Adaptation of Serine Hydroxymethyltransferase to Low Temperatures. Int. J. Biol. Macromol. 46, 37–46 (2010). 19815026 10.1016/j.ijbiomac.2009.09.009

[b31] Angelucci F. . The Crystal Structure of Archaeal Serine Hydroxymethyltransferase Reveals Idiosyncratic Features Likely Required to Withstand High Temperatures. Proteins. 82, 3437–3449 (2014). 25257552 10.1002/prot.24697

[b32] Bhatt A. N., Khan M. Y. & Bhakuni V. The C-terminal Domain of Dimeric Serine Hydroxymethyltransferase Plays a Key Role in Stabilization of the Quaternary Structure and Cooperative Unfolding of Protein: Domain Swapping Studies with Enzymes Having High Sequence Identity. Protein Sci. 13, 2184–2195 (2004). 15273312 10.1110/ps.04769004 PMC2279811

[b33] Paiardini A., Gianese G., Bossa F. & Pascarella S. Structural Plasticity of Thermophilic Serine Hydroxymethyltransferases. Proteins. 50, 122–134 (2003). 12471605 10.1002/prot.10268

[b34] Kirino H. . Hydrophobic Interaction at the Subunit Interface Contributes to the Thermostability of 3-Isopropylmalate Dehydrogenase From an Extreme Thermophile, Thermus Thermophilus. Eur J Biochem. 220, 275–281 (1994). 8119295 10.1111/j.1432-1033.1994.tb18623.x

[b35] Larkin M. A. . Clustal W and Clustal X Version 2.0. Bioinformatics. 23, 2947–2948 (2007). 17846036 10.1093/bioinformatics/btm404

[b36] Penn O. . GUIDANCE: A Web Server for Assessing Alignment Confidence Scores. Nucleic Acids Res. 382, W23–W28 (2010). 10.1093/nar/gkq443 PMC2896199 20497997

[b37] Trivedi V. . Crystal Structure of Binary and Ternary Complexes of Serine Hydroxymethyltransferase From Bacillus Stearothermophilus: Insights Into the Catalytic Mechanism. J. Biol. Chem. 277, 17161–17169 (2002). 11877399 10.1074/jbc.M111976200

[b38] Kawashima S. . AAindex: Amino Acid Index Database, Progress Report 2008. Nucleic Acids Res. 36, D202–D205 (2008). 17998252 10.1093/nar/gkm998 PMC2238890

[b39] Horimoto K., Suzuki H. & Otsuka J. Discrimination Between Adaptive and Neutral Amino Acid Substitutions in Vertebrate Hemoglobins. J. Mol. Evol. 31, 302–324 (1990). 2124278 10.1007/BF02101125

[b40] Phillips J. C. . Scalable Molecular Dynamics with NAMD. J. Comput. Chem. 26, 1781–1802 (2005). 16222654 10.1002/jcc.20289 PMC2486339

[b41] MacKerell A. D. . All-Atom Empirical Potential for Molecular Modeling and Dynamics Studies of Proteins. J. Phys. Chem. B. 102, 3586–3616 (1998). 24889800 10.1021/jp973084f

[b42] Van der Spoel D. . GROMACS: Fast, Flexible, and Free. J. Comput. Chem. 26, 1701–1718 (2005). 16211538 10.1002/jcc.20291

[b43] Humphrey W., Dalke A. & Schulten K. VMD: Visual Molecular Dynamics. J. Mol. Graph. Model. 14, 33–38 (1996). 10.1016/0263-7855(96)00018-5 8744570

