# Supplementary material for: Determinants of Thermostability in Serine Hydroxymethyltransferase Identified by Principal Component Analysis
Source: Sci Rep. 2017 Apr 19;7:46463. doi: 10.1038/srep46463 (PMC5396068; doi:10.1038/srep46463)
Supplement: Supplementary Information [file srep46463-s1.pdf]

Determinants of Thermostability in Serine Hydroxymethyltransferase Identified by Principal Component Analysis

Fei Leng, Lu-Yun Wu, Chang Lu and Xian-Ming Pan*

From the Key Laboratory of Bioinformatics, Ministry of Education, School of Life Sciences, Tsinghua University, Beijing, 100084, China

*To whom correspondence should be addressed: Prof. Xian-Ming Pan, School of Life Sciences, Tsinghua University, Beijing, 100084, China

Tel: +86-10-62792827; Fax: +86-10-62792827

E-mail: pan-xm@mail.tsinghua.edu.cn

position	MRT
1	-0.01025
2	0.009362
3	0.009362
4	0.009362
5	-0.00742
6	-0.00415
7	-0.00415
8	-0.01766
9	-0.05821
10	0.012807
11	-0.07852
12	-0.20014
13	-0.42378
14	0.150327
15	0.150327
16	0.133987
17	0.058824
18	0.058824
19	0.065359
20	0.062092
21	0.075163
22	0.078431
23	0.22549
24	0.248366
25	0.313725
26	0.330065
27	-0.09822
28	-0.12418
29	-0.12418
30	-0.09804
31	-0.07843
32	-0.06863
33	0.429606
34	0.58691
35	0.610228
36	0.613054
37	0.686275
38	-0.11305
39	0
40	0
41	0
42	0
43	0

44	0
45	0
46	0
47	0.013514
48	0
49	0
50	0
51	0
52	0
53	0.175676
54	0
55	0
56	0
57	0
58	0
59	0
60	0
61	0
62	0
63	0
64	0
65	0
66	0
67	0
68	0
69	0
70	0
71	0
72	0
73	0
74	0
75	0
76	0
77	0
78	0
79	0
80	0
81	0
82	0
83	0
84	0
85	0
86	0
87	0

88	0
89	0
90	0
91	0
92	0
93	0
94	0
95	0
96	0
97	0
98	0
99	0
100	0
101	0
102	0
103	0
104	0
105	0
106	0
107	0
108	0
109	0
110	0
111	0
112	0
113	0
114	0
115	0
116	0
117	0
118	0
119	0
120	0
121	0
122	0
123	0
124	0
125	0
126	0
127	0
128	0
129	0
130	0
131	0

132	0
133	0
134	0
135	0
136	0
137	0
138	0
139	0.013514
140	0
141	0.026144
142	0.022876
143	0.022876
144	0.022876
145	0.022876
146	0.022876
147	0.022876
148	0.022876
149	0.022876
150	0.022876
151	0.022876
152	0.022876
153	0.022876
154	0.022876
155	0.022876
156	0.022876
157	0.022876
158	0.022876
159	0.022876
160	0.022876
161	0.022876
162	0.022876
163	0.022876
164	0.022876
165	0.022876
166	0.022876
167	0.022876
168	0.022876
169	0.022876
170	0.022876
171	0.022876
172	0
173	0
174	0
175	0

176	0
177	0
178	0
179	0
180	0
181	0
182	0
183	0
184	0
185	0
186	0
187	0
188	0
189	0.013072
190	-0.00654
191	0
192	0
193	0
194	0
195	0
196	0
197	0
198	0
199	0.006536
200	0
201	0
202	0
203	0
204	0
205	0
206	0
207	0
208	0
209	-0.04902
210	-0.21569
211	-0.04575
212	0
213	0
214	0
215	0
216	0
217	0
218	0
219	0

220	0
221	0
222	0
223	0
224	0
225	0
226	0
227	0
228	0
229	0
230	0
231	0
232	0
233	0
234	0
235	0
236	0
237	0
238	0
239	0
240	0
241	0
242	0
243	0
244	0
245	0
246	0
247	0
248	0
249	0
250	0
251	0
252	0
253	0
254	0
255	0
256	0
257	0
258	0
259	0
260	0
261	0
262	0
263	0

264	0
265	0
266	0
267	0
268	0
269	0
270	0
271	0
272	0
273	0
274	0
275	0
276	0.029412
277	0.022876
278	0.022876
279	0
280	0
281	0
282	0
283	0
284	-0.43243
285	0
286	0
287	0
288	0
289	0
290	0
291	0
292	0
293	0
294	0
295	0
296	0
297	0
298	0
299	0
300	0
301	0
302	0
303	0
304	0
305	0
306	0.256757
307	0.368574

308	-0.51351
309	-0.39189
310	-0.41892
311	-0.24324
312	-0.05662
313	-0.12374
314	-0.23529
315	0.026144
316	0.022876
317	0.022876
318	0.022876
319	0.022876
320	0.022876
321	0.006536
322	0.006536
323	0.022876
324	0.022876
325	0.022876
326	0.022876
327	0.022876
328	0.006536
329	0.022876
330	0.022876
331	0.333333
332	-0.26144
333	-0.04204
334	-0.02288
335	0.862745
336	0.663399
337	0
338	0
339	0
340	0
341	0
342	0
343	0
344	0
345	0
346	0
347	0
348	0
349	0
350	0
351	0

352	0
353	0
354	0
355	0
356	0
357	0
358	0
359	0
360	0
361	0
362	0
363	0
364	0
365	0
366	0
367	0
368	0
369	0.425896
370	0
371	0
372	0
373	0
374	0
375	0
376	0
377	0
378	0
379	0
380	0
381	0
382	0
383	0
384	0
385	0
386	0
387	0
388	-0.53595
389	0.754902
390	0
391	-0.45946
392	-0.44595
393	-0.01351
394	-0.01351
395	-0.44595

396	0.013514
397	0.006536
398	0.081699
399	0.081699
400	0.088235
401	0.088235
402	-0.02941
403	0
404	0
405	0
406	0
407	0
408	0
409	0
410	0
411	0
412	0
413	0
414	0
415	0
416	0
417	0
418	0
419	0
420	0
421	-0.8381
422	0.918301
423	-0.02765
424	-0.01351
425	-0.06757
426	-0.06757
427	-0.08108
428	0.277248
429	-0.51131
430	0.094771
431	0.653595
432	0
433	-0.00654
434	-0.00654
435	-0.00654
436	-0.00654
437	-0.00654
438	0
439	0

440	0
441	0
442	0
443	0
444	0
445	0
446	0
447	0
448	0
449	0
450	0
451	0
452	0
453	0
454	0
455	0
456	0
457	0.009804
458	0
459	0.081081
460	-0.35135
461	0
462	0
463	0
464	0
465	0
466	0
467	0
468	0
469	0
470	0
471	0
472	0
473	0
474	0
475	0
476	0
477	0
478	0
479	0
480	0
481	-0.00327
482	-0.00327
483	0

484	0
485	0
486	0
487	0
488	0
489	0
490	0
491	0
492	0
493	0
494	0
495	0
496	0
497	0.0643
498	0.061032
499	0.026409
500	0.122946
501	-0.52703
502	-0.12162
503	-0.12162
504	-0.12162
505	-0.12162
506	-0.12162
507	-0.32132
508	0.205882
509	0.271242
510	0.267974
511	0.196078
512	0.196078
513	0.183007
514	0.022876
515	0.019608
516	0.022876
517	0.022876
518	0.022876
519	0.022876
520	0.022876
521	0.029412
522	0.029412
523	0.431373
524	-0.04028
525	-0.05882
526	-0.04902
527	-0.02288

528	-0.0098
529	0
530	0
531	0
532	0
533	0
534	0
535	0
536	-0.00327
537	0
538	0
539	0.010246
540	0.010246
541	0.010246
542	0.006978
543	0.006978
544	0.008744
545	-0.2557
546	-0.30118
547	-0.44303
548	-0.3593
549	-0.51095
550	-0.50963
551	-0.51572
552	-0.43791
553	-0.38624
554	-0.37926
555	-0.17241
556	0.608108
557	0.662162
558	0.662162
559	0.702703
560	0.716216
561	0.743243
562	0.756757
